# Protective Effects of Swertiamarin against Methylglyoxal-Induced Epithelial-Mesenchymal Transition by Improving Oxidative Stress in Rat Kidney Epithelial (NRK-52E) Cells

**DOI:** 10.3390/molecules26092748

**Published:** 2021-05-07

**Authors:** Kirti Parwani, Farhin Patel, Dhara Patel, Palash Mandal

**Affiliations:** Department of Biological Sciences, P. D. Patel Institute of Applied Sciences, Charotar University of Science and Technology, Changa 388421, India; kirtiparwani@gmail.com (K.P.); farhinpatel1993@gmail.com (F.P.); dharapatel.phd@gmail.com (D.P.)

**Keywords:** advanced glycation end product (AGE), oxidative stress, epithelial to mesenchymal transition, AGE-inhibitor, swertiamarin, diabetic nephropathy

## Abstract

Increased blood glucose in diabetic individuals results in the formation of advanced glycation end products (AGEs), causing various adverse effects on kidney cells, thereby leading to diabetic nephropathy (DN). In this study, the antiglycative potential of Swertiamarin (SM) isolated from the methanolic extract of *E. littorale* was explored. The effect of SM on protein glycation was studied by incubating bovine serum albumin with fructose at 60 °C in the presence and absence of different concentrations of swertiamarin for 24 h. For comparative analysis, metformin was also used at similar concentrations as SM. Further, to understand the role of SM in preventing DN, in vitro studies using NRK-52E cells were done by treating cells with methylglyoxal (MG) in the presence and absence of SM. SM showed better antiglycative potential as compared to metformin. In addition, SM could prevent the MG mediated pathogenesis in DN by reducing levels of argpyrimidine, oxidative stress and epithelial mesenchymal transition in kidney cells. SM also downregulated the expression of interleukin-6, tumor necrosis factor-α and interleukin-1β. This study, for the first time, reports the antiglycative potential of SM and also provides novel insights into the molecular mechanisms by which SM prevents toxicity of MG on rat kidney cells.

## 1. Introduction

Type 2 Diabetes (T2D) is a metabolic syndrome, which results due to peripheral insulin resistance, affecting both metabolism and the disposal of glucose. The occurrence of diabetes is accelerating worldwide, with a consequent increase in diabetes associated complications like diabetic nephropathy (DN), neuropathy, retinopathy, and cardiovascular complications. Hyperglycaemia is one of the main reasons for causing DN, and various clinical trials have established that the progression to DN can be slowed and also reversed by good and strict glycemic controls [1,2,3]. Elevated blood glucose, which induces reactive oxygen species (ROS) formation, is understood to be one of the main reasons for the formation of advanced glycation end products (AGEs) in the intracellular and extracellular environment [4]. AGEs are the products of nonenzymatic glycation between free amino acids and reducing sugar via the Maillard reaction resulting into yellowish-brown fluorescent and insoluble adducts.

The formation of AGEs is divided into two stages. In the earlier stage, Amadori product, a stable compound is formed from an unstable Schiff base [5]. Amadori products can either form reactive dicarbonyls like glyoxal and methylglyoxal (MG) or undergo various chemical reactions like condensation, dehydration and oxidation to form AGEs. The mechanism and the different stages involved in the formation of AGEs are explained in Supplementary Materials Figure S1. These adducts can alter the normal physiological functions of a protein upon glycation [6]. This can alter the half-life of the proteins and affect their physiological clearance. Patients with persistent T2D are established to have vastly higher levels of AGEs [7]. The role of AGEs in DN has been documented by reports suggesting the negative correlation between the accumulation of AGEs in plasma and renal function [8]. AGEs bind to their receptor called receptor for advanced glycation end products (RAGE) and primarily lead to the generation of ROS and oxidative stress which further leads to the damage of the renal tubular cells and mesangial cells leading to DN. Further, AGEs lead to an imbalance between synthesis and degradation of extracellular matrix components like collagen and cause their accumulation in the mesangium, tubular interstitial cells and glomerulus basement membrane leading to their hardening, which is a hallmark feature of DN [9].

Reduced antioxidant defenses have also been reported in diabetic patients thereby providing acceleration to the development of chronic complications [10]. It is reported that AGEs could alter the physiological functions of an antioxidant enzyme like superoxide dismutase (SOD) and could inactivate it [11]. Glycation has been found to induce aggregation and structural modifications in catalase by targeting lysine residues [12]. This reduced antioxidant machinery along with increased ROS due to hyperglycaemia can be detrimental for a cell. Many synthetic inhibitors that can restrain the formation of AGEs have been identified and studied but they have been withdrawn back due to their lower potency and side effects. Therefore, there arises a rationale to identify some inhibitor, which along with being potent and effective, should have low toxicity.

Traditional and herbal therapies are gaining importance slowly over conventional medicines due to their advantage of having lower or no side-effects. The molecules present in the plants are shown to be hypoglycaemic, hypolipidemic, and antioxidant in nature. Compounds in the plants like phenolics [13], polysaccharides [14] and carotenoids [15], etc. are shown to possess antiglycative properties. In addition, the regular consumption of edible products rich in antioxidants and polyphenolic compounds could be beneficial in the prevention of diabetes and its related complications [16]. *Enicostemma littorale* commonly called Mamejava has been shown to possess a hypoglycaemic effect in T2D rats [17]. *E. littorale* contains important phytoconstituents like gentianine, enicoflavine, gentiocrucine, swertiamarin (SM), etc., to name a few. SM has been shown to possess anticholinergic [18], antihyperlipidaemic [19], hypoglycaemic [20] and antioxidant effects [21]. SM in combination with quercetin, ameliorated T2D and oxidative stress in streptozotocin-treated Wistar rats [22].

Since glycation is a process associated with increased free radical formation, compounds with antioxidant activities could also act as an inhibitor for the formation of AGEs [23]. The antiglycation potential of SM has not been explored yet, although there exists a report where *Swertia Chirayata* extract, which contains various active molecules along with SM, showed inhibition of fructose mediated glycation. Given the important role of SM in diabetes mellitus and its antioxidant potential, the present study was executed to understand and assess its antiglycation potential. To our knowledge, this is the first report that shows inhibition of fructose mediated glycation by SM and also unravels the molecular mechanism involved in its protective role in MG-induced damage on kidney cells, thereby preventing DN.

## 2. Results

### 2.1. Isolation of SM from E. littorale and Characterization Using High-Performance Liquid Chromatography (HPLC), Fourier-Transform Infrared Spectroscopy (FTIR) and Liquid Chromatography-Mass Spectra(LC-MS)

**Figure 1:**
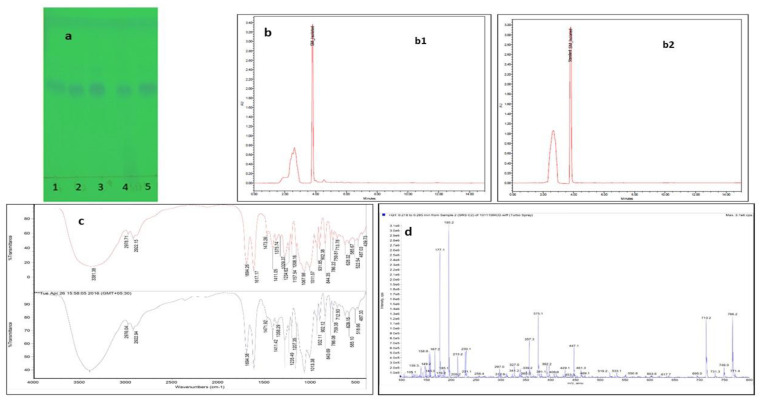
(**a**) TLC profile: Lane 1, 2: Fractions containing SM along with impurities. Lane 3: Standard Lane 4: Methanolic extract of *E. littorale* Lane 5: Fraction containing SM in the TLC mobile phase (Chloroform: methanol in 8:2 *v/v* proportion) (**b**) The HPLC chromatogram of SM isolated in the lab (**b1**) and of the standard SM (**b2**) at 1 mg/mL using acetonitrile: water (10:90) as the mobile phase. (**c**) An overlay of FT-IR spectrum of standard SM (red) and SM isolated in the lab (blue). (**d**) The mass spectrum (LC-MS) of isolated swertiamarin showing characteristic *m/z* of 375.1.

Using the solvent chromatography on silica, SM was successfully isolated from *E. littorale* as confirmed by TLC. The presence of SM was confirmed with reference to the standard SM using the chloroform: methanol (8:2 *v/v*) as a mobile phase with R_f_ of 0.58. The visualization was done at 254 nm as shown in Figure 1a. By our isolation method, we report 7% yield of SM from the methanolic extract of *E. littorale*, which to our knowledge is the highest yield obtained until now. To analyze the purity of the isolated sample, the HPLC of the isolated compound was done and was compared with the standard SM. The HPLC profiles of the isolated SM (a) and the standard (b) are shown in Figure 1b. Both the standard and the lab isolated SM gave a characteristic peak at 238 nm, which is the absorbance maxima of SM as reported earlier. The retention time of the lab isolated SM was found to be 3.801 which fairly matched to 3.805 of that of the standard with the acetonitrile: water (10:90 *v/v*) as the mobile phase.

The analysis of the different functional groups present in the isolated sample was done using FTIR and was also compared with the standard SM. The lab isolated SM fairly matched the FTIR spectrum of the standard SM as shown in Figure 1c. FTIR spectra of both, isolated and standard swertiamarin showed definite peaks between wavenumbers 4000–500 cm^−1^. The characteristic O-H stretch peak was found at 3381 cm^−1^, C-H stretch at 2292 cm^−1^, C=O stretch at 1694 cm^−1^, C=C stretch at 1617 cm^−1^, C-O-C at 1411 cm^−1,^ and C=CH_2_ stretch at 844 cm^−1^.This validates the purity of the isolated compound as the wavenumbers for both compounds (lab isolated SM and standard) match with each other.

The validation of the molecular weight of the isolated SM was done using LC-MS. The LC-MS spectrum of SM reported earlier showed a molecular ion peak at *m/z* of 374, and LC-MS spectrum for SM isolated in our lab as shown in Figure 1d, showed *m/z* of 375.1 in positive ion mode, indicating the addition of H^+^ to the compound, thereby increasing molecular ion peak by 1, as shown in Merck Index. Together the characterization data using different methods confirmed the isolated compound to be SM and by our isolation method, we report 7% yield of SM from the methanolic extract of *E. littorale*, which to our knowledge is the highest yield obtained until now (Figure 1).

### 2.2. SM Inhibits the Formation of AGEs

**Figure 2:**
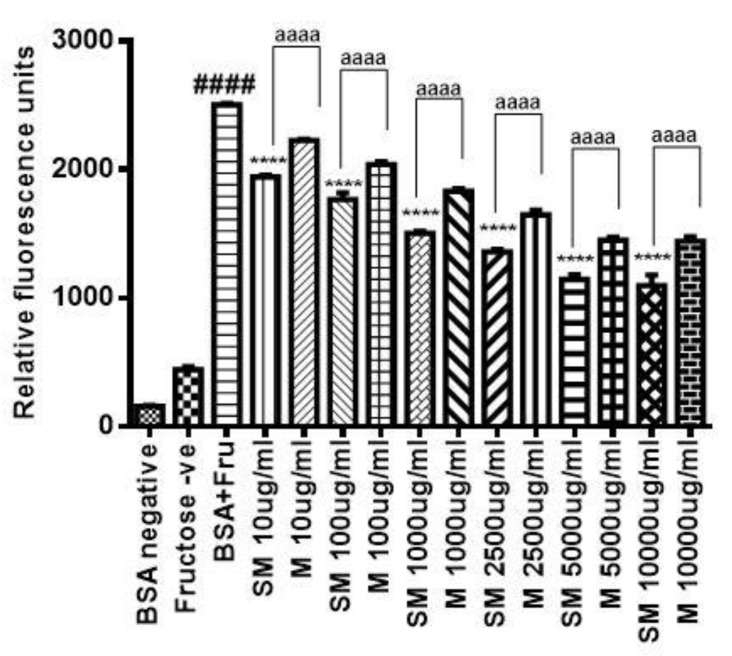
The intensity of fluorescence of AGEs in the presence and absence of swertiamarin (SM) and its comparison with metformin (M) at different concentrations measured using an excitation wavelength of 370 nm and an emission wavelength of 440 nm. The results are Mean ± SD of 3 individual experiments. ^####^ *p* < 0.0001 represents comparison between bovine serum albumin (BSA) negative and BSA + Fructose (Fru) group. **** *p* < 0.0001 represents the statistical significance w.r.t the BSA + Fru group. ^aaaa^ *p* < 0.0001 represents the comparison between SM and M.

The nonenzymatic glycation of the proteins leads to the formation of AGEs. AGEs possess characteristic property of fluorescence after glycation. The formation of AGEs was analyzed by checking the fluorescence at excitation and emission wavelengths of 370 nm and 440 nm, respectively. The fluorescence intensity was used as a measure of glycation, i.e., higher the intensity of fluorescence, the higher is the glycation of proteins. The intensity of fluorescence was significantly decreased (*p* ≤ 0.0001) in SM treated samples as compared to untreated samples that contained neither SM nor metformin. In addition, SM could inhibit the formation of AGEs better than metformin (*p* < 0.0001) at similar doses (Figure 2). This indicates that SM could be a better glycation inhibitor and therefore can prevent the formation of endogenous AGEs due to persistent hyperglycaemia in diabetic subjects.

### 2.3. SM Reduces Fructose Mediated Hyperchromicity

**Figure 3:**
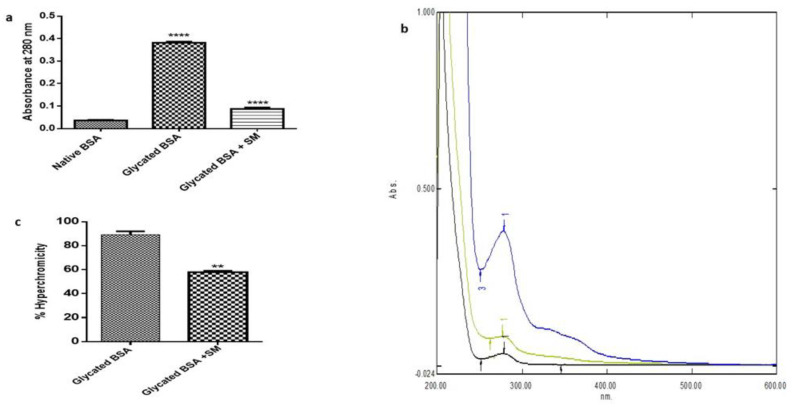
Antiglycative effect of swertiamarin at 100 µg/mL (SM 100) as shown by absorbance at 280 nm using UV-spectrophotometer (**a**), the structural changes as seen by the shift in the peaks due to glycation (**b**), suggesting hyperchromicity (**c**). The results are Mean ± SD of 3 individual experiments. **** *p* < 0.0001, ** *p* < 0.054241.

Glycation of the proteins results in the structural changes of the proteins, which modifies their characteristic UV absorption spectrum. The changes in the structure of BSA and its absorbance were additionally analyzed by spectrophotometer (Figure 3a). As shown in Figure 3b, compared to normal BSA (Black line), glycated BSA (Blue line) shown increased absorbance and hyperchromicity (89%) due to fructose mediated changes in the structure of bovine serum albumin (BSA). SM could prevent the fructose mediated changes in the BSA (Green line) as depicted by reduced hyperchromicity (57.9%) indicating the inhibition of glycation (*p* < 0.0054) (Figure 3c).

### 2.4. SM Reduces Carbonyl Content in the Glycated BSA

**Figure 4:**
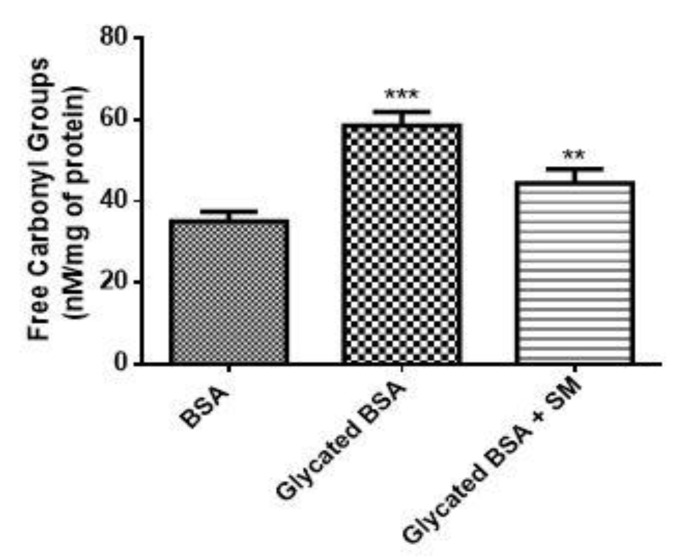
Estimation of carbonyl content. As compared to the native BSA, glycated BSA showed remarkably higher level of carbonyl content. The treatment with SM showed reduction in the carbonyl content, as compared to the glycated BSA. (*** *p* < 0.001 and ** *p* < 0.01). Results are mean ± SD of 3 individual experiments.

The carbonyl content was analysed as a marker for oxidation of protein during glycation. As shown in Figure 4, the glycated BSA showed remarkably higher carbonyl content upon the reaction with fructose, indicating the formation of radicals and dicarbonyls via Maillard reaction (*** *p* < 0.001). The treatment with SM could significantly limit the formation of carbonyls, which is due to its ability to scavenge free radicals as an antioxidant (** *p* < 0.01).

### 2.5. SM Prevents Fructose Mediated Side-Chain Modifications in BSA

**Figure 5:**
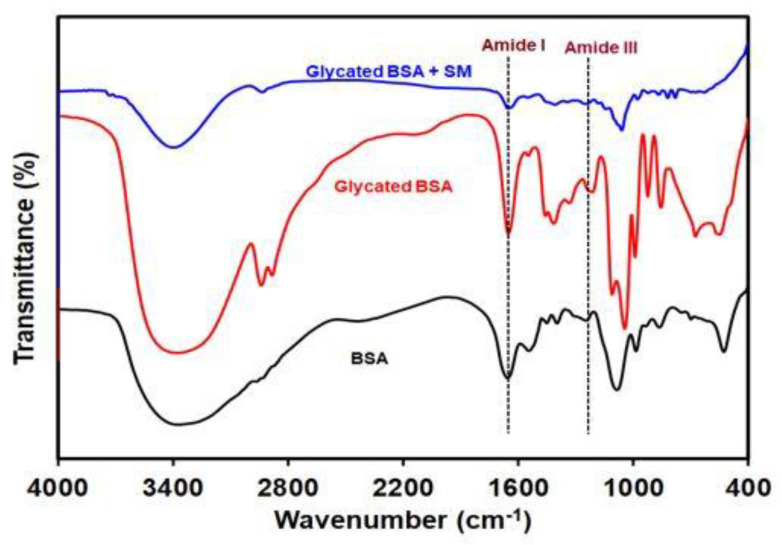
FTIR spectra of BSA (black line), glycated BSA (red line) and the glycated BSA treated with SM (blue line).

The modifications in the functional group in the side chains of the BSA was analysed using FTIR. As shown in Figure 5, FTIR spectrum of the native BSA showed a peak at 1655 cm^−1^ corresponding to amide I, while red-shift was observed in case of glycated samples, where the amide I shifted to 1651 cm^−1^. The treatment with SM showed a minor shift, indicating the relatively stable BSA molecule, as the amide I shift indicates the presence of α-helical structure in the protein. The amide III band shifted 9 cm^−1^, from 1245 cm^−1^ to lower wavenumber 1236 cm^−1^ in the glycated BSA as compared to native BSA. However, the treatment of glycated BSA with SM did not show major shift in the wavenumber compared to native BSA with wavenumber 1242 cm^−1^. Together these results indicate that glycated BSA exhibited various side-chain modifications, affecting the structure of BSA upon glycation. Table 1 shows the different bands and wavenumbers obtained in all the samples.

### 2.6. Effect of MG and SM on the Viability of NRK-52E Cells

**Figure 6:**
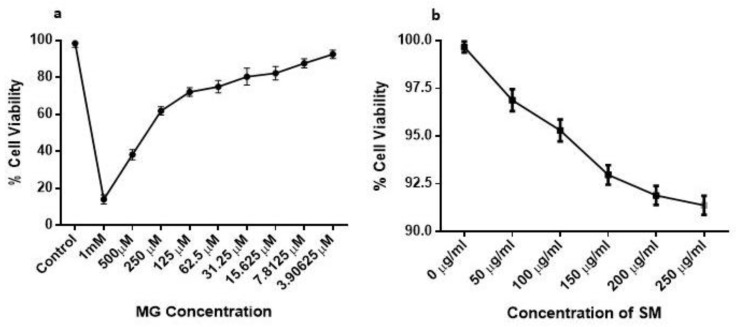
(**a**) The effect MG on the cell viability in NRK-52E cells. NRK-52E cells when treated with different concentrations of MG caused cell death in a dose-dependent manner as compared to control. (**b**) The cytotoxicity of SM was checked on NRK-52E cells. The treatment with SM did not show any toxicity on the NRK-52E cells at various concentrations. Results are Mean ± SD of 3 individual experiments.

The toxicity of MG on NRK-52E cells was analyzed for fixing the dose for further experiments on NRK-52E. The treatment with MG induced cell death of NRK-52E and the dose-dependent decrease in cell viability was observed with an increase in the concentration of MG (*p* < 0.0001) (Figure 6a). To determine the dose of MG to be used for further experiments with NRK-52E, we determined the IC50 value for MG, which was found to be 200 μM and therefore the cells were challenged with 200 μM MG for testing the protective effects of SM in presence of MG. However, SM did not show any toxicity on NRK-52E cells at all the different concentrations used for the experiment (Figure 6b).

### 2.7. SM Improves the Morphology of the NRK-52E Cells and Ameliorates against MG-Induced Damage

**Figure 7:**
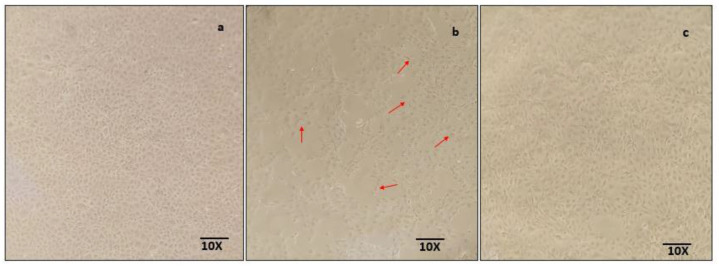
Morphology of NRK-52E cells in each treatment group at 24 h. The untreated NRK-52E cells showed its characteristic epithelial morphology (**a**). The treatment with 200 µM MG changed the epithelial morphology of NRK-52E cells to elongated fibroblast like morphology, indicated with arrows (**b**). Cotreatment with 100 µg/mL SM prevented MG—induced morphological changes in NRK-52E cells (**c**). Above images are representative microscopy images of each group under 10× objective.

The effect of MG on the morphology of NRK-52E cells can be seen in Figure 7. MG treatment on the cells changed the cobblestone morphology of NRK-52E cells to a round and elongated fibroblast-like shape, indicating the cellular damage due to MG, which was prevented when the cells were treated with MG along with SM. The morphological changes are indicative of various inflammatory changes occurred due to the presence of MG, which was ameliorated by SM, as seen by the intact epithelial morphology of NRK-52E cells.

### 2.8. SM Alleviates the Formation of Argpyrimidine Like AGEs in NRK-52E Cells

**Figure 8:**
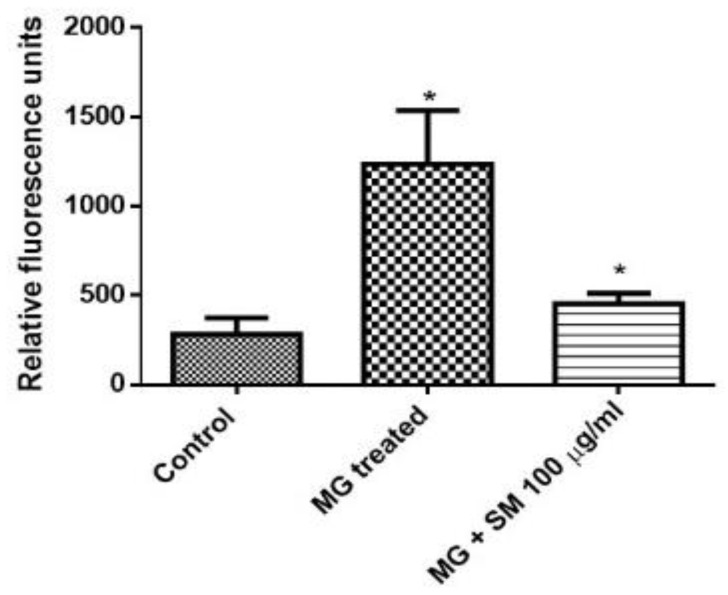
Detection of argpyrimidine levels. As compared to the control group, treatment with 200 µM MG in the NRK-52E cells after 24 h resulted in the formation of argpyrimidine by MG-induced modifications of arginine which could be prevented in MG and 100 µg/mL SM cotreatment group (* *p* < 0.05). Results are Mean ± SD of 3 individual experiments.

Dicarbonyls like MG react with arginine in the proteins to form AGEs. Argpyrimidine is a major modification of amino acid arginine by MG. As shown in Figure 8 NRK-52E cells when treated with MG showed higher levels of argpyrimidine as compared to the untreated cells. The treatment of SM along with MG alleviated the levels of argpyrimidine which could be due to the inhibition of modification of arginine by MG, thereby reducing the glycation induced damage to the cells.

### 2.9. SM Alleviates the Formation of MG—Induced ROS and Mitigates the Oxidative Stress in NRK-52E Cells

**Figure 9:**
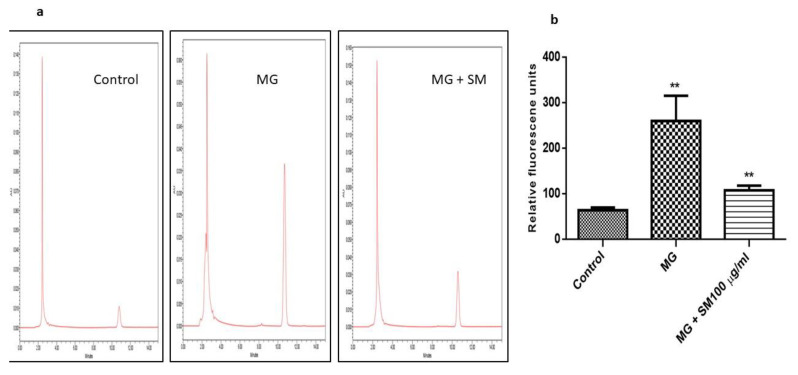
(**a**) HPLC chromatograms of MDA measured from NRK-52E cells after DNPH derivatization. The treatment with 200 µM MG in NRK-52E cells increased the levels of MDA measured using ODS2 reverse phase column in the presence of acetonitrile and milliQ water containing 0.2% acetic acid, with a ratio 38:62 respectively as the mobile phase. Treatment with 100 µg/mL SM in the presence of MG attenuated the production of MDA. (**b**) The levels of ROS were also measured using fluorescence spectroscopy with excitation and emission wavelengths 495 nm and 529 nm, respectively. Treatment with SM in the presence of MG could inhibit the elevation of ROS significantly (** *p* < 0.01) as shown in MG treated group (** *p* < 0.01), proving the antioxidative characteristic of SM. Results are Mean ± SD of 3 individual experiments.

The treatment with MG on NRK-52E cells resulted in the elevated oxidative stress, which could be due to the increased levels of AGEs formation in the presence of MG. Increased oxidative stress leads to the peroxidation of lipids resulting in the formation of malondialdehyde (MDA), a marker for oxidative stress in the cells. MG treatment in the NRK-52E cells exacerbated the levels of MDA as measured by HPLC (Figure 9a). This was ameliorated by the treatment of SM as seen by the reduced MDA levels. Oxidative stress further increased the formation of ROS in the cells, measured through fluorescence spectroscopy (Figure 9b). Treatment with SM in the presence of MG, prevented the formation of ROS, thereby unraveling the role of SM in preventing the MG—induced oxidative stress.

### 2.10. SM Alleviates MG—Induced ER Stress in NRK-52E Cells

**Figure 10:**
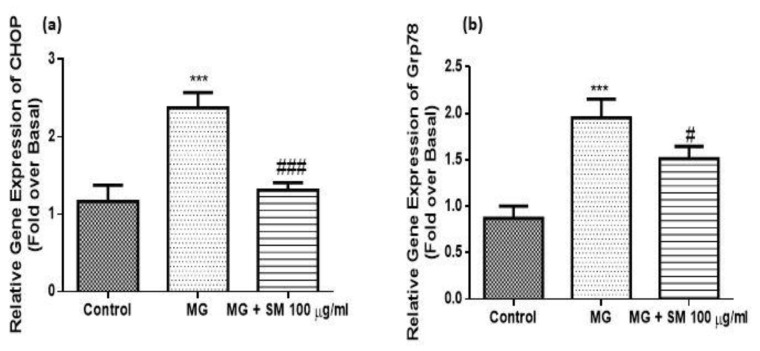
mRNA expression of ER stress genes (**a**) CHOP and (**b**) Grp-78 using qRT-PCR. The expression of CHOP and Grp78 was significantly upregulated in MG-exposed cells as compared to the untreated control NRK-52E cells after 24 h. The treatment with 100 µg/mL SM along with 200 µM MG could alleviate the upregulation of CHOP and Grp78, indicating the protection against ER stress. Results are Mean ± SD of 3 individual experiments analysed by ANOVA. The symbol *** *p* < 0.001 indicates the comparison of MG group w.r.t control and ^#^ *p* < 0.05, ^###^ *p* < 0.001 indicates the comparison of SM group w.r.t. MG group.

The treatment of the NRK-52E cells with MG leads to the production of AGEs which further results in the upregulation of ER stress by activating the unfolded protein response (UPR). The mRNA expression of ER stress markers like CCAAT-enhancer-binding protein homologous protein (CHOP) and glucose regulatory protein 78 (Grp78) were upregulated in the MG-treated NRK-52E cells. The treatment with SM along with MG could alleviate the upregulation of CHOP and Grp78, indicating the protection against ER stress (Figure 10).

### 2.11. Treatment with SM Improves the Inflammatory Response in MG Treated NRK-52E Cells

**Figure 11:**
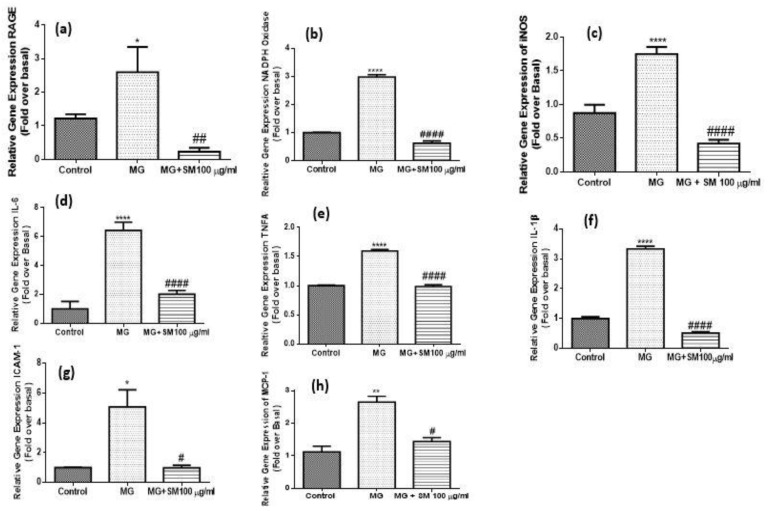
qRT-PCR analysis results of (**a**) RAGE (**b**) NADPH oxidase (**c**) iNOS (**d**) IL-6 (**e**) TNF-α (**f**) IL-1β (**g**) ICAM-1, and (**h**) MCP-1 in MG-treated NRK-52E cells alone and SM for 24 h. The gene expression levels were calculated after normalizing against housekeeping 18S rRNA and are presented as relative mRNA expression units. Values represent mean ± SD of 3 individual experiments. The symbol * *p* < 0.05, ** *p* < 0.01, **** *p* < 0.0001 indicate the comparison of MG group w.r.t control and ^#^ *p* < 0.05, ^##^ *p* < 0.01, ^####^ *p* < 0.0001 indicate the comparison between MG and SM group.

Cells when treated with 200 µM MG caused the upregulation of the mRNA levels of RAGE, that could be due to elevated levels of AGEs in MG-treated NRK-52E cells. The interaction of RAGE with AGEs and MG alone induced oxidative stress which resulted in the upregulation of ROS via increased NADPH oxidase levels in NRK-52E cells. The oxidative stress and the upregulated expression of RAGE induced the expression of various proinflammatory cytokines like interleukin-6 (IL-6), tumor necrosis factor-α (TNF- α), interleukin-1β (IL-1β) and inducible nitric oxide synthase (iNOS) in the cells treated with MG as compared to untreated control NRK-52E cells. This further exacerbated the expression of cell adhesion molecules like intracellular cell adhesion molecule-1 (ICAM-1) and monocyte chemoattractant protein-1 (MCP-1) in NRK-52E cells, leading to inflammation and cellular injury to kidney cells. Nonetheless, SM treatment at a concentration of 100 µg/mL to MG exposed cells (Figure 11) could prevent the upregulation of all the inflammatory cytokines under investigation (^####^ *p* < 0.0001; ^###^ *p <* 0.001; ^##^ *p* < 0.01), therefore explaining the preventive role of SM in inflammation of kidney cells.

### 2.12. SM Improves MG-Induced Epithelial Mesenchymal Transition (EMT) in NRK-52E Cells

**Figure 12:**
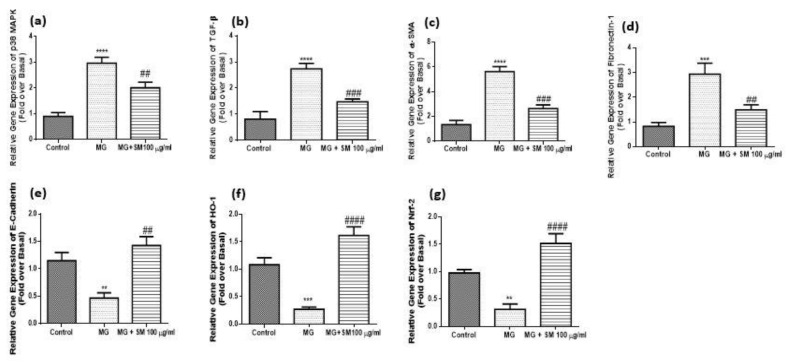
qRT-PCR analysis results of (**a**) p38 MAPK (**b**) TGF-β (**c**) α-SMA (**d**) Fibronectin-1 (**e**) E-cadherin (**f**) HO-1 and (**g**) Nrf-2 in MG-treated NRK-52E cells alone and SM for 24 h. The gene expression levels were calculated after normalizing against housekeeping 18S rRNA and are presented as relative mRNA expression units. Values represent mean ± SD of 3 individual experiments. The symbol ** *p* < 0.01, *** *p* < 0.001, **** *p* < 0.0001 indicate the comparison of MG group w.r.t control and ^##^ *p* < 0.01, ^###^ *p* < 0.001, ^####^ *p* < 0.0001, indicate the comparison between MG and SM group.

**Figure 13:**
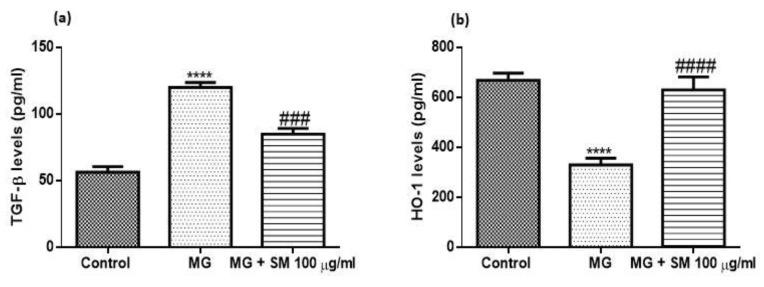
Protein levels of TGF-β and HO-1 in NRK-52E measured using ELISA. The treatment with 200 μM MG for 24 h, up-regulated the levels of TGF-β proteins which was prevented by the co-treatment with 100 μg/mL SM. The levels of HO-1 were declined in the MG treated group after 24 h which was alleviated by SM, proving the role of SM in inhibiting TGF-β expression by upregulating HO-1. Values represent mean ± SD of 3 individual experiments. **** *p* < 0.0001 indicate the comparison of MG group w.r.t control and ^###^ *p* < 0.001, ^####^ *p* < 0.0001, indicate the comparison between MG and SM group.

NRK-52E cells when treated with MG, showed morphological changes and the epithelial nature of cells changed to elongated, and fibroblast-like phenotype (Figure 10), possibly due to epithelial-mesenchymal transformation (EMT). SM treated group, did not show any change in the epithelial morphology of NRK-52E cells. MG induces the upregulation of p38 MAPK and also the downstream signaling by activating the transcription factors leading to various molecular changes in the kidney cells. It is previously reported that transforming growth factor- β (TGF-β) plays a very crucial role in the pathogenesis of DN. TGF-β induces the upregulation of fibroblast markers like alpha-smooth muscle actin (α-SMA), and fibronectin-1, along with downregulation of epithelial marker E-cadherin. Treatment with MG showed upregulated expression of these markers in NRK-52E cells which was ameliorated in the cells cotreated with MG and SM. These results indicate that MG induces the EMT changes in epithelial NRK-52E cells (Figure 12). As oxidative stress plays a very important role in the development of renal fibrosis via EMT, we also analyzed the levels of key intracellular antioxidant machinery like nuclear factor (erythroid-derived 2)-like 2 (Nrf-2) and heme oxygenase-1 (HO-1) expression levels. The treatment with MG in NRK-52E cells significantly attenuated the levels of Nrf-2 and HO-1, which were induced by the treatment with SM. Together these results indicate that SM ameliorated EMT in NRK-52E cells by inhibiting the expression of TGF-β via upregulation of HO-1 and Nrf-2 (Figure 12). As shown in Figure 13, MG treatment upregulated the protein levels of TGF-β and decreased the levels of HO-1, which was alleviated by SM treatment.

### 2.13. SM Shows Better Affinity and Binding with RAGE as Compared to Argpyrimidine

**Figure 14:**
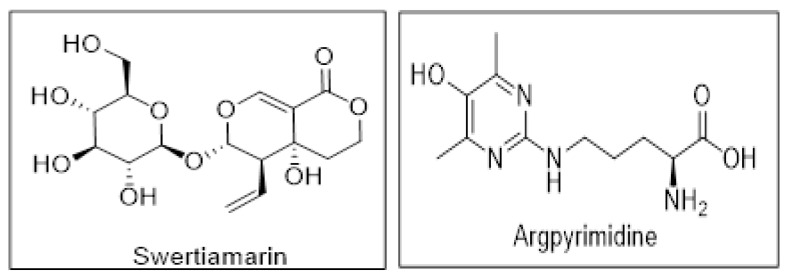
Chemical structure of swertiamarin and Argpyrimidine.

AGEs interact with their cellular receptor RAGE and initiate the inflammatory pathways by production of proinflammatory cytokines which further results in the oxidative stress. The selective inhibition of AGE-RAGE axis by preventing the binding of AGEs with RAGE can inhibit the deleterious effects observed due to their interaction. Therapeutic molecules that can bind with RAGE, thereby blocking the binding of AGEs with RAGE can be used as an approach to attenuate the progression of DN. In our study we have shown that SM not only reduces the expression of RAGE but also reduces the levels of AGEs like Argpyrimidine which is formed in the presence of MG. Therefore, to understand the possible role and mechanism of how SM prevents the MG-induced damage on NRK-52E cells, we adopted an in silico approach, where we checked if SM could interact with RAGE so as to prevent the binding of AGEs and therefore inhibit the activation of AGE-RAGE axis. Therefore, we docked both SM and Argpyrimidine with RAGE to check the affinity of both the ligands with RAGE. A total of 13 different molecules that encompassed different states of SM and Argpyrimidine were generated by LigPrep module. All the ligands and their various states were docked in the upstream C-Domain Type 1 using the Glide XP module. Further, the molecules were quantitatively evaluated based on their scores and qualitatively based on the type of interactions made with the residues of the amino acids. The chemical structures of the ligands are shown is Figure 14.

**Figure 15:**
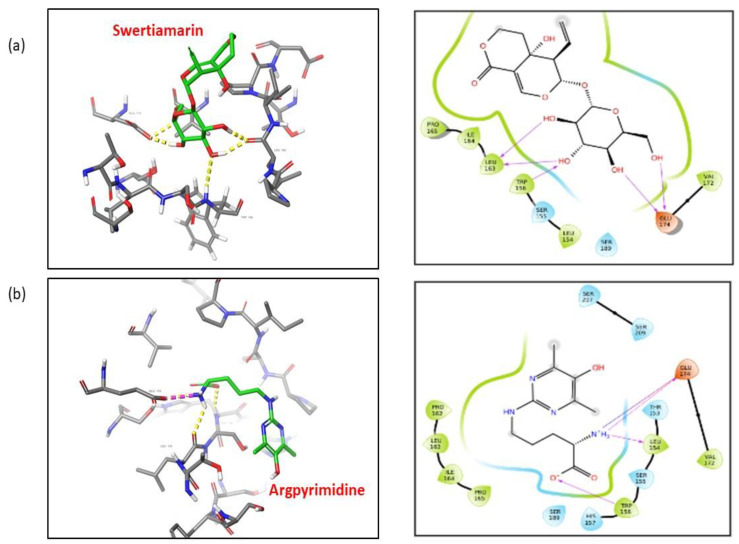
(**a**) Binding of SM with RAGE (left) and the amino acids involved in the interaction (right panel). (**b**) Binding of Argpyrimidine with RAGE (left) and the amino acid involved in the interaction (right panel).

SM showed a G score of −4.7 kcal/mol while the docking score was −4.7 kcal/mol. The interactions formed by this ligand are as follows: OH acts as hydrogen bond acceptor for Leu 163 and Glu 174, and as a hydrogen bond donor to the amino acid Trp 156 (Figure 15a). Argpyrimidine on the other hand showed G score of −2.75 kcal/mol while the docking score is −2.73 kcal/mol. The interactions formed by this ligand are as follows: O^−^ acts as hydrogen bond acceptor for TRP 156, and hydrogen from amino group as a hydrogen bond donor to the amino acids Leu 154 and Glu 174. Further, the protonated nitrogen of argpyrimidine forms a salt bridge with GLU 174 (Figure 15b). Table 2 summarizes different interactions between SM-RAGE and Argpyrimidine-RAGE docking.

## 3. Discussion

In the present study, we report isolation of SM and its role in inhibiting the formation of AGEs. The SM was isolated in the lab from *E. littorale* with better yield (7%) by column chromatography, which to our knowledge is the highest yield using silica of 60–120 mesh size as compared to the 5% yield obtained using silica based column chromatography [24], 0.6% using sephadex LH-20 column [25] and 2% using centrifugal partition chromatography [26]. The absorbance maxima of isolated SM matched with the absorbance maxima of the SM isolated by Rana et al. in the HPLC [26] and with the UV spectral analysis by Vishwakarma et al. to be at 238 nm [24,27]. The molecular ion peak of the isolated SM was 375.1 which matched with the results obtained in the literature along with their FT-IR fingerprint, proving the isolated compound to be SM [28]. Owing to its various properties like anticholinergic [18] antihyperlipidaemic [19] and given the important role of SM in diabetes mellitus [20] and its antioxidant potential [29], the present study was executed to understand and assess its antiglycation potential.

The persistent hyperglycaemia in the blood leads to the production of ROS which results in the oxidative stress. Oxidative stress results not only due to excessive ROS but may also result due to the defects or impairment in the antioxidant enzymes which otherwise can clear the ROS [30]. Increased blood glucose exacerbates the formation of ROS and is one of the prime reasons for AGEs formation, which further leads to various complications of T2D like DN [31,32,33]. Free radicals formed due to higher glucose levels can induce the formation of AGEs from Amadori products [34]. This is explained by the study which shows the increased levels of MG in the blood of diabetics as compared to nondiabetic individuals [35] and also supported by a study proving the accumulation of AGEs in most DN patients [36]. Under diabetic conditions, the excess of glucose leads to the activation of polyol pathway, resulting in the formation of fructose [37]. Fructose leads to the formation of AGEs and ROS by promoting the glycation of proteins and lipids, also aided by MG which is a dicarbonyl formed during glycolytic pathway for glucose metabolism [38]. Hence, preventing the glycation induced by fructose and MG could be one of the prime strategies to inhibit the formation of AGEs and prevent the diabetes related complications [39]. We, therefore assessed the potential of SM in inhibiting the fructose induced glycation. Fructose mediated glycation of proteins increases their fluorescence intensity at 440 nm which is due to formation of AGEs [40]. In the extant study, BSA glycated with fructose showed increased fluorescence intensity as compared to BSA treated with fructose in the presence of SM, which explains the role of SM in being able to prevent the formation of AGEs. To understand the potency of SM in being able to prevent the formation of AGEs, we compared it with metformin which is already known to prevent MG mediated glycation of albumin and also prevent renal tubular injury [41,42]. SM at similar dose to that of metformin could significantly inhibit the formation of AGEs much better as compared to metformin, which proves it to a better glycation inhibitor than metformin. The glycation of the BSA results in the increase in the carbonyl content, indicative of oxidative changes in the BSA [43]. In the present study, in accordance with the literature, we found increased carbonyl content in the glycated BSA. The treatment of SM in presence of glycated BSA, could reduce the carbonyl content, which possibly can be attributed to the antioxidative potential of SM.

The glycation induced by fructose leads to the modifications of various proteins, which renders the loss of function of proteins. This can be understood by hyperchromicity that can be attributed to the loss or fragmentation of the proteins, which causes the exposure of the aromatic amino acids responsible for its higher absorption at 280 nm [44]. It is of prime importance to understand the changes in the proteins caused due to their frutosylation, as the concentration of fructose increases up to as high as 5 mM in the kidneys and peripheral nerves due to polyol pathway [45] The structural changes as understood by hyperchromicity could be due to side chain modifications in the proteins due to glycation. Such modifications lead towards the structural as well as functional group modifications in the proteins, which can be analysed by FT-IR. We found the changes in the peaks corresponding to amide I from 1655 cm^−1^ of the native BSA to 1651 cm^−1^ in the glycated BSA. The treatment with SM showed a minor shift, indicating the relatively stable BSA molecule. The amide I band is a characteristic feature attributed to α-helix in the protein, and the decrease in the wavenumber indicates loss of α-helix in the glycated BSA, and therefore the structural change in the BSA due to glycation. Similarly, the amide III band of native BSA shifted from 1245 cm^−1^ to lower wavenumber 1236 cm^−1^ in the glycated BSA as compared to native BSA and the treatment of glycated BSA with SM did not show major shift in the wavenumber compared to native BSA with wavenumber 1242 cm^−1^. The pronounced effect on shifting of amide III wavenumbers could be due to increase in the β-sheet and β-turns in the glycated BSA sample [46]. The treatment with SM however prevented the structural changes in the BSA as indicated by minor shift, suggesting the preventive role of SM in inhibiting the glycation induced structural changes in the protein. Our results show that the fructose mediated glycation was prevented when the glycation of BSA by fructose was carried out in presence of SM, as seen by reduced hyperchromicity which indicates the role of SM in preventing the fructose or AGEs mediated damage to kidney cells.

As discussed earlier, AGEs lead to the formation of ROS, which results in the oxidative stress. The formation of ROS and the repair of the damage caused due to ROS and oxidative stress is normally rescued in the cells by its antioxidant enzyme machinery comprising of antioxidant enzymes like catalase and SOD. However, glycation of enzymes like catalase and SOD lead to the loss of their activity, rendering an imbalance in homeostasis of oxidants and antioxidants in the cell [47]. ROS being toxic to kidney cells promotes cell death by inflammatory and fibrogenic reactions in the kidney cells [48]. Therefore, to understand the cellular effects of AGEs and how SM could prevent AGEs mediated progression of DN, we used an in vitro MG-induced model of DN. The concentration of MG to be used in the study was determined using MTT based cell viability assay. The dose-dependent decrease in the cell viability was observed, possibly because MG with increasing concentration may have induced apoptosis in the cells. Since nearly 50% cell death was observed at 199.5 µM concentration of MG, we chose 200 µM MG concentration for our further experiments, which was supported by the studies on rat mesangial cells, where 200 µM MG resulted in apoptosis of mesangial cells after 8 h of incubation with MG [49].

MG in particular can act as a source to the formation of a specific fluorescent AGE called Argpyrimidine, which is formed by a reaction of MG with the guanidine group of arginine [38]. Argpyrimidine was also found to be accumulated in the intima and media of small arteries of the kidneys of diabetic patients, which suggested its role in the progression of DN [50]. Approximately two to three-fold higher levels of argpyrimidine are found in diabetic patients as compared to nondiabetic individuals. The argpyrimidine levels also are found to positively correlate with glycosylated hemoglobin [51]. Higher levels of argpyrimidine were found to be present in the mesangial cells of rat kidney cultured in high glucose-containing medium [52]. Our study shows that SM can prevent the formation of AGEs and specifically could also inhibit the formation of MG mediated argpyrimidine formation in NRK-52 E cells. We also found increased levels of ROS in MG treated cells which as discussed earlier could be possibly due to the formation of AGEs [34]. However, the cells treated with SM along with MG showed reduced ROS, suggesting the antiglycative property of SM.

It is well established that oxidative stress and increased free radical can induce lipid peroxidation. This is because lipids are most vulnerable to the attack by ROS and reactive nitrogen species (RNS) [53]. This can generate increased levels of MDA, which is one of the major mutagenic and toxic products formed during the process of lipid peroxidation [54]. The present study shows that cotreatment of NRK-52E cells with MG and SM reduced the levels of ROS which led to reduced oxidative stress as evidenced by decreased production of MDA, which otherwise was higher due to lipid peroxidation in MG treated NRK-52E cells. Chronic hyperglycaemia, if unmanaged, not only culminates into various complications but also leads to pancreatic β-cell death. In a recent study, it was shown that MG leads to β-cell death by the formation of AGEs [55].

The MG mediated AGEs could readily interact with their membrane-bound receptor RAGE and result in the kidney dysfunction by inducing chronic inflammation. In a study on OVE26 mouse, it was shown that RAGE deletion could prevent renal function in diabetic mice, thereby explaining the crucial role played by RAGE in DN [56]. Therefore, elucidating the role of SM in MG-mediated RAGE expression became crucial, and we found that SM could decrease the mRNA expression of RAGE as compared to that of MG-treated cells. The activation of RAGE due to AGEs can lead to various inflammatory reactions as discussed earlier, by inducing the expressions of inflammatory markers like NADPH oxidase, TNF-α, IL-1β, IL-6, which gets accelerated due to the expression of cellular adhesion molecules like ICAM-1 [57]. In the current study, we found that treatment of MG not only induced the up regulated expression of proinflammatory cytokines like TNF-α, IL-1β, IL-6 but also of NADPH oxidase and ICAM-1, which was ameliorated in the cells treated with SM along with MG. The interaction of RAGE with AGEs can lead to the generation of ROS via NADPH oxidase which can promote the expression of TGF-β via NFκB, mitogen-activated protein kinase (MAPK), or PKC pathways in mesangial and renal tubulointerstitial cells in the kidneys [58]. We in the current study showed the MG mediated upregulation of p38 MAPK, which by further activation of inflammatory cascades led to the increase in the pro-inflammatory markers, causing the progression towards DN.

TGF-β being a pro-fibrotic cytokine plays a very crucial role in the renal fibrosis via EMT. EMT remains a very important phenomenon in the pathogenesis of DN, as it induces the expression of pro-fibrotic markers and mesenchymal markers like fibronectin 1 and α-SMA, inducing the tubulointerstitial fibrosis [59,60]. In addition, inhibition of particular stages involved in EMT, reduced the formation of fibrotic lesions in the kidney and therefore EMT is being considered to be significant in the development of DN [61,62,63]. We found that MG exposure changed the epithelial morphology of NRK-52E to more extended, fibroblast-like morphology, possibly due to higher mRNA expression of TGF-β as compared to lower expression of TGF-β in the cells co-treated with SM and MG. In the current study, MG induced the expression of fibronectin 1 and α-SMA along decreasing the epithelial marker e-cadherin, confirming the MG mediated EMT. These changes were prevented by SM. Our results are in agreement with a previous study, where TGF-β led to fibrosis of renal proximal tubules cells, leading to the death of the cells [64].

AGEs are known to induce pro-inflammatory signaling culminating in oxidative stress, further inducing apoptosis in the nephrons leading to renal fibrosis and therefore DN [65]. It is well established that ROS and oxidative stress plays a very important role in development of renal complications including EMT [66]. Therefore, the cellular antioxidants could be chosen as one of the prime targets for inhibiting EMT. The role of cellular antioxidant machinery like Nrf-2 and HO-1 has been demonstrated by investigations which showed that Nrf2^−/−^ mice were highly sensitive to high glucose induced oxidative damage, and the HO-1 deficiency is associated with increased fibrosis and increased tubular expression of TGF-β [67,68]. In our study, we found that MG induced downregulation of HO-1 and Nrf-2 was upregulated in SM treated cells.

To understand the possible mechanism by which SM inhibited the expression of RAGE, we explored the in silico approach adopted by researchers [69] to analyze the role of SM in binding with RAGE, thereby blocking the binding of AGEs with RAGE. As MG leads to the production of Argpyrimidine, we docked both Argpyrimidine and SM with RAGE to check for their binding energies. Argpyrimidine is known to bind in the C-Domain type 1 present in the RAGE; therefore, we chose the region ranging from 123 to 219 amino acids for performing the docking [69]. SM showed better binding energy and docking score as compared to Argpyrimidine, which suggests that RAGE binds with more preference with SM than Argpyrimidine, thereby blocking the binding of Argpyrimidine with RAGE. The difference in binding energy is −1.95 kcal/mol. This docking result is complementary to the experimental results obtained in vivo, which also shows that binding of Swertiamarin results in reduction of the AGEs and the expression of its receptor RAGE.

In conclusion, we report that SM inhibits binding of AGEs with RAGE and hence prevents the activation of RAGE signaling and p38 MAPK, thereby inhibiting the downregulation of HO-1 and Nrf-2 to prevent the EMT in rat kidney cells.

## 4. Materials and Methods

Bovine serum albumin (BSA, fraction V) was acquired from Himedia (Mumbai, India). Potassium bromide (FTIR grade) was obtained from Sigma-Aldrich (St. Louis, MO, USA). Acetonitrile (HPLC grade), HPLC grade water for HPLC; hexane (AR grade), ethyl acetate (AR grade) and methanol (AR grade) were acquired from Merck (Darmstadt, Germany) for the isolation of SM using column chromatography. All the solvents were used in their pure form without any preprocessing.

### 4.1. Isolation of SM Using Silica Chromatography

The dry plant *E.littorale* was acquired from Saurashtra region, Gujarat, India. The plant was press dried and then powdered in a crusher. The powder was immersed overnight in double the volume of 70% methanol (SRL, Mumbai, India) and 30% water. The process was repeated three times for a single batch and then filtered. The filtrate was vacuum evaporated using a Heidolph rotary evaporator (Schwabach, Germany), to get a dry hydroalcoholic extract.

To obtain SM from the hydroalcoholic extract, silica based column chromatography was done. Furthermore, 1 g of hydroalcoholic extract was coated with 3 g silica. The extract was chromatographed using silica gel (60–120 mesh size, Merck, Germany) with hexane and then with hexane containing ethyl acetate (60–97%), followed by ethyl acetate and then with ethyl acetate mixed with methanol in the order of increasing polarity (0.5–1.5%). Fractions with different polarities were assessed for the presence of SM by thin-layer chromatography (TLC) using the solvent system of chloroform: methanol (8:2). The presence of SM was established by co-chromatography of the standard SM (TCI, Japan) along with different fractions. Fractions with SM were pooled and dried. They were further purified by precipitating the methanol dissolved residue with nonpolar solvents like diethyl ether (yield: 7%).

### 4.2. Characterization of Isolated SM for Its Purity

The characterization as well as the purity of the isolated compound was confirmed by various methods which included TLC, HPLC, LC-MS and FTIR.

#### 4.2.1. Characterization Using High Performance Liquid Chromatography (HPLC)

For HPLC analysis, the solution of SM isolated in the lab was prepared to achieve a final concentration of 1 mg/mL in methanol. The samples and solvents to be used as mobile phases were filtered through 0.2 µm syringe filters (Axiva, India). HPLC was carried out using the previously described mobile phase for SM with slight modifications by using acetonitrile: water (10:90) as the mobile phase [70]. The flow rate was 1 mL/min and the column temperature was maintained at 25 °C. The detection wavelength and mode for SM was 238 nm and photodiode detector (PDA).

#### 4.2.2. Characterization Using Fourier-Transform Infrared Spectroscopy (FTIR) and Mass Spectrometer (MS)

The isolated compound was analyzed by FTIR for the identification of functional groups present in the compound. The standard was also used as a reference for the isolated compound. A homogenous solution of both standard and the compound was individually prepared in potassium bromide (KBr) and subjected to FTIR spectrophotometer (Thermofisher Scientific, Waltham, MA, USA). The compound isolated was also subjected to positive-ion Electrospray Ionization, i.e., ESI using Perkin-Elmer Applied Biosystem Sciex API 2000 (Waltham, MA, USA) for identification of its characteristic molecular ion peak.

### 4.3. Analysis of Antiglycative Potential of SM Using In Vitro Glycation Assay

Antiglycative studies were done as suggested by McPherson et al. with slight modifications [71]. Bovine Serum Albumin (BSA, Himedia, Mumbai, India) and Fructose (Merck, Darmstadt, Germany) were used to induce glycation. In brief, in a 1.5 mL eppendorf, 100 µL of 60 mg/mL BSA (20 mg/mL final concentration) was incubated with 100 µL of 1.5 M fructose (0.5 M final concentration) and 100 µL of potassium phosphate buffer (pH 7.4). Negative control was incorporated as well, which consisted of 100 µL of 60 mg/mL BSA (20mg/mL final concentration) and 200 µL of potassium phosphate buffer (pH 7.4). To understand the antiglycative potential of the compound, the same reaction was carried out in the presence of varying concentrations of SM (1 µg/mL to 10 µg/mL). For the comparative studies, metformin was used as a positive antiglycative control at the same concentrations as of SM. The reaction was incubated at 60 °C for 24 h and samples were analyzed for fluorescence due to AGEs on a Perkin Elmer LS-55 spectrofluorometer (Waltham, MA, USA) by using an excitation wavelength of 370 nm and an emission wavelength of 440 nm.

### 4.4. Analysis of Protein Modifications Due to Glycation by UV Absorbance Spectroscopy

The native, glycated BSA and samples in which BSA was glycated in presence of SM were analyzed for their absorption spectra on Shimadzu UV–1700 spectrophotometer (Kyoto, Japan) in 200 to 800 nm wavelength range using a quartz cuvette. The samples were analyzed for the hyperchromic shift due to glycation by fructose as suggested by Allarakha et al. [45].

### 4.5. Estimation of Protein Carbonyl Content

The analysis of the carbonyl content as a marker for oxidative damage to protein due to glycation was done according to the protocol suggested by Meeprom et al. [43]. From each group, 100 μL of the sample was taken and mixed with 400 μL of 10 mM 2,4-dinitrophenylhydrazine (DNPH) prepared in 2.5 M HCl solution, followed by the incubation for 1 h in the dark. After the incubation, the protein was precipitated on ice using 500 μL of 20% *w/v* tri-choloroacetic acid (TCA) solution and subjected to centrifugation at 10,000× *g* for 10 min at 4 °C. The protein in the pellet was washed thrice with 500 μL of 1:1 solution of ethanol and ethyl acetate followed by resuspension in 250 μL of 6 M guanidium hydrochloride solution. The absorbance was recorded at 370 nm and the carbonyl content was measured using the molar extinction coefficient of DNPH, i.e., ε = 22,000 M^−1^ cm^−^^1^. The results were expressed as nM carbonyl content/mg of protein.

### 4.6. Fourier Transform Infrared Spectroscopy of the Protein Samples

To check and analyze the functional and conformational changes in the BSA due to fructose mediated glycation, FTIR spectroscopy was done as suggested by Liu et al. [46]. The samples were lyophilized and then mixed with FTIR grade KBr in 1: 100 ratio (sample: KBr) to make a homogenous mixture and pressed to prepare a transparent pellet. The pellet was subjected to analysis in the transmission mode in the range of 400–4000 cm^−1^ on FTIR spectrophotometer (Thermofisher Scientific, Waltham, MA, USA).

### 4.7. Culturing of NRK-52 E Cells

Normal Rat Kidney (NRK-52E) cell line was acquired from National Centre for Cell Sciences (NCCS), Pune, India. The cells were cultured in Dulbecco’s Modified Eagle Medium Low Glucose (5.5 mM/L) medium with 10% fetal bovine serum, 1% L-glutamine, and 1% penicillin/streptomycin (Thermofisher Scientific, Waltham, MA, USA) at 37 °C in a 5% CO_2_ incubator. The cells were grown till 80–90% confluency, after which they were used for experiments with MG in the presence and absence of SM.

#### Cell Viability and Dose Determination of MG and SM Using MTT Assay

The concentration of MG (Sigma-Aldrich, St. Louis, MO, USA) used during the experiments was determined by checking MG toxicity on NRK-52E cells using 3-(4,5-dimethylthiazol-2-yl)-2,5-diphenyltetrazolium bromide (MTT) assay described by Riss et al. [72]. In brief, 1 × 10^4^ cells were seeded in a 96-well plate. After 24 h, cells were exposed to different concentrations MG (1 mM, 500 µM, 250 µM, 125 µM, 62.5 µM, 31.25 µM, 15.625 µM, 7.812 µM, and 3.906 µM), along with an untreated control well, for 24 h. Similarly, the toxicity of SM was checked on NRK-52E. NRK-52E cells were treated with different concentrations of SM (50 µg/mL, 100 µg/mL, 150 µg/mL, 200 µg/mL and 250 µg/mL) along with a group left untreated with SM for 24 h. After treating them for 24 h, 10 µL of MTT solution was added to each well at a final concentration of 0.5 µg/mL and the cells were incubated at 37 °C in a 5% CO_2_ atmosphere for 3 h in the dark. Following the incubation period, formazan crystals formed in each well were solubilized using 100 µL dimethyl sulfoxide (DMSO) solution. The absorbance was then measured at 570 nm to determine the viability of cells.

### 4.8. MG Stimulation and Different Treatment Groups

To check the effect of MG on NRK-52E cells, 8 × 10^5^ cells were seeded in a 60 mm culture plates followed by incubation for 24 h. After this, cells received different treatments based on the respective groups like control (only growth medium), MG (medium containing 200 µM MG) and MG + SM 100 µg/mL (medium containing 200 µM MG in the presence of 100 µg/mL SM). The effect of MG-induced stress on kidney cells was assayed by checking the ROS production, lipid peroxidation, argpyrimidine levels and transcript levels of various genes involved in the progression of DN by qRT PCR.

### 4.9. Estimation of Argpyrimidine

To estimate the levels of Argpyrimidine, NRK-52E cells (8 × 10^6^ cells/well) were seeded onto 60mm plates, and the cells were incubated for 24 h. After 24 h, cells were treated with MG, in the presence and absence of SM for 24 h. After the incubation, the cells were lysed using the lysis buffer followed by centrifugation at 16,000 rpm for 10 min at 4 °C. The supernatant was then analyzed using a Hitachi F-7000 fluorescence spectrophotometer (Tokyo, Japan) with an excitation wavelength of 330 nm and an emission wavelength of 380 nm for the presence of Argpyrimidine [51].

### 4.10. Estimation of Reactive Oxygen Species (ROS)

In order to estimate oxidative stress caused by MG in the presence or absence of SM, the cells were treated with 200 μM MG alone or along with SM and incubated for 24 h. After treatment, cells were incubated with 5(6)-carboxy-20,70-dichlorofluorescein Diacetate (Carboxy-H2-DCFDA) (Sigma-Aldrich, St. Louis, MO, USA) in the dark with a final concentration of 30 μM at 37 °C for 1 h. The cells were then harvested, washed with PBS, and resuspended in PBS. ROS production in the cells was measured by measuring the fluorescence of the sample at an excitation wavelength—485 nm and an emission wavelength—530 nm, using a fluorescence spectrophotometer (Perkin Elmer LS-55, Waltham, MA, USA [73].

### 4.11. Estimation of MDA as a Measure of Lipid Peroxidation by HPLC

Total malondialdehyde (MDA) in the cultured cells was estimated using HPLC by following the method described by Tukozkan et al. with some modifications [74]. Summarily, the medium was removed from the plates and the cells were rinsed with PBS. Cells were homogenized in cold 1.15% KCl to make 10% homogenate. 500 µl of the homogenate was then mixed with 100 µl of 6 M NaOH, and the samples were incubated in a water bath at 60 °C for 45 min. The hydrolyzed sample was then acidified with 250 µL of 35% perchloric acid. The samples were subjected to centrifugation at 15,000× *g* for 10 min. After centrifugation, 250 µL of the supernatant was collected and mixed with 25 µL of DNPH solution, followed by 10 min incubation in the dark. The samples were then analyzed by HPLC in an ODS2 reverse column using acetonitrile: water (38:62) containing 0.2% acetic acid as a mobile phase. Isocratic conditions were maintained during HPLC with a flow rate of 1 mL/min and the MDA was detected in the samples at 310 nm with the UV detector with a retention time of about 10 min. The concentration of MDA was detected in the sample by comparing it with the standard curve prepared using 1,1,3,3 tetraethoxypropane.

### 4.12. Isolation of RNA, cDNA Synthesis and Analysis of Gene Expressions of Various Markers by qRT-PCR

Total RNA was isolated from the control and treated cells using TRIzol reagent. The purity and concentration of RNA in the sample were measured at 260 nm and A260/A280 ratio of the sample was determined using NanoDrop 2000 (Thermofisher Scientific, Waltham, MA, USA). A total of 1 μg RNA was used to synthesize cDNA using first strand cDNA synthesis kit. For qPCR, maxima SYBR Green/ROX qPCR master mix was used to quantify the mRNA expression levels for all genes under investigation on the Agilent Strategene Mx3005P system. The qRT-PCR system comprised of 1 μL cDNA, 0.5 μL of 10 μM FP (forward primers), 0.5 μL of 10 μM RP (reverse primers), 10 μL SYBR Green Mastermix, and 8.0 μL milli-Q water in a 20 μL reaction system. qRT-PCR cycling conditions were set according to Kema, V.H. et al. 2017 [75]. The 2^-ΔΔCt^ method (fold change over basal) was applied to evaluate mRNA expression levels in both control and treated cells. 18S rRNA was presented as an internal reference gene control. The list of primer sequences sets used are tabulated in Table 3.

### 4.13. Estimation of Protein Levels of TGF-β and HO-1 by ELISA

The protein levels of TGF-β and HO-1 were analysed from the cultured NRK-52E cells after the treatment with 200 μM MG in the absence and presence of 100 μg/mL SM. Briefly, the cultured cells from different groups were removed using cell scraper after washing thrice with 1X PBS. The cells were then centrifuged at 1500 rpm for 10 min under cooling conditions. The collected cell free supernatant was analyzed as per the manufacturer’s protocol (Abcam, Cambridge, UK). The levels of TGF-β and HO-1 were measured in pg/mL.

### 4.14. Molecular Docking Studies

#### 4.14.1. Homology Modelling

Receptor for Advanced Glycation End Products (RAGE) from *Rattus norvegicus* consists of 402 amino acids [76]. The FASTA sequence for building up the 3D model was obtained from UniProt database (UniProt ID: Q63495) [77]. The 3D model of RAGE was constructed using RAGE (*Mus musculus*) (PDB ID: 4IM8) as the template as it showed a sequence identity of 92.04% [76,78]. The 3D model was created using SWISS-MODEL server which uses homology modelling method, wherein it aligns the amino acid sequence of the desired protein with the protein that has closest identity to our desired protein and whose structure has been solved, thus developing a model for the unknown desired protein whose sequence is known. The generated model was further used for docking studies. The molecular docking was performed using Glide module (XP) of Maestro software (Schrodinger) version 12.7.156 (New York, NY, USA) [79].

#### 4.14.2. Protein Preparation Protocol

The protein structure of Receptor for Advanced Glycation End Products (RAGE) from *Rattus norvegicus* was constructed using SWISS-MODEL server. The structure so generated needs to be prepared using protein preparation wizard, which helps in pre-processing, optimizing hydrogen bonds and minimizing the protein, from its raw state to a better state which can be useful for the further calculations. The protein was preprocessed by assigning bond order, adding the missing hydrogen atoms, and converting the selenomethionine to methionine. The missing side chains and loops were filled in using Prime module, which is the protein refinement application. The termini were capped with N-acyl and N-methyl groups and finally, the water molecules which were more than 5 Å away from the het groups were deleted. A pH of 7.0 ± 2.0 was set in Epik to account for the possible tautomeric state of amino acid residues. The hydrogen bonds were optimized with PROPKA at a pH of 7. Lastly, the RAGE protein model was minimized using optimized potentials for liquid simulations 4 (OPLS4) force field [80,81,82].

#### 4.14.3. Receptor Grid Generation

Receptor grid generation forms the first step of molecular docking protocol. A volume of the active site is represented by the generated grid file. The ligands of interest are then docked in this grid. Initially, the minimized protein was selected to generate the grid. It has been reported in the literature that Argpyrimidine binds to the upstream C-Domain Type 1 in RAGE [69]. Hence, the amino acids from 123 to 219, which represent the upstream C-Domain Type 1, were selected for generating the grid for docking SM and Argpyrimidine. The centroid was generated around these amino acids, and further criteria were set to default, to generate the grid for docking [80,81,82].

#### 4.14.4. Glide Molecular Docking and Analysis of Docking

Glide is the molecular docking module in Maestro. It has a widespread use from knowing the ideal interactions between a particular ligand and the protein to screening large libraries of molecules for high throughput screening. The molecular docking using Glide starts with procuring the grid file and the Ligprep output file. Further, XP mode was selected for better accuracy and further criteria were set as default. The docking module was run and results were obtained and analysed using XP visualizer in Schrodinger [80,81,82].

#### 4.14.5. Statistical Analysis

All respective experiments were performed at least thrice. Data of the replicates were calculated as mean ± SD. GraphPad Prism 7 software (GraphPad Software Inc., California Corporation, San Diego, CA, USA) was used to analyze the results. The differences between all groups were analyzed using one way analysis of variance (ANOVA). Variations between groups were considered to be significant at *p* values less than or equal to 0.05.

## 5. Conclusions

The present study depicted the protective effect of SM in inhibiting the formation of fructose induced AGEs and its role in ameliorating the progression of DN. SM could prevent the fructose mediated conformational changes in the BSA. The formation of AGEs like argpyrimidine from MG upon interaction with RAGE led to the increased production of ROS and therefore resulted in the generation of oxidative stress. Moreover, treatment with SM in the presence of MG-treated NRK-52E cells prevented the oxidative stress and inflammation, by upregulating the antioxidants level of Nrf-2 and HO-1, thereby reducing the activation of TGF-β and prevented against the MG-induced EMT changes in NRK-52E. We for the first time report here that the inhibition of the of RAGE/ MAPK/ TGF-β pathway could be a possible mechanism contributing towards protecting effects of SM in preventing the DN induced changes in NRK-52E cells in presence of MG. In addition, the current study for the first time demonstrates that the molecular interactions between RAGE and SM can inhibit the binding of Argpyrimidine and block the AGE-RAGE axis, which also is supported by the cellular studies. Additionally, this may provide strong evidence for an in vivo study as a future prospect to unravel the mechanism of SM in inhibiting AGEs induced DN.

## Figures and Tables

**Table 1:** Comparative analysis of bands and wavenumbers in all samples.

Samples	Bands and Wavenumber (cm^−1^)	
	Amide I	Amide III
Native BSA	1655	1245
Glycated BSA	1651	1236
Glycated BSA + SM	1654	1242

**Table 2:** Interaction of ligands, type of interaction and total binding energies of RAGE with SM and Argpyrimidine.

Sr. No.	Interaction	Point Interaction	Donor Atom	Acceptor Atom	Type of Interaction	Bond Distance (Å)	Energy Binding (G Score) (kcal/mol)
**1**	RAGE: Swertiamarin	Trp 156–Swertiamarin	Trp 156: H	Swertiamarin: O	Hydrogen Bond	2.62	−4.7
		Leu 163–Swertiamarin	Swertiamarin: H	Leu 163: O	Hydrogen Bond	1.90	
		Leu 163–Swertiamarin	Swertiamarin: H	Leu 163: O	Hydrogen Bond	1.90	
		Glu 174–Swertiamarin	Swertiamarin: H	Glu 174: O	Hydrogen Bond	2.23	
		Glu 174–Swertiamarin	Swertiamarin: H	Glu 174: O	Hydrogen Bond	1.75	
**2**	RAGE: Argpyrimidine	Leu 154–Argpyrimidine	Argpyrimidine: H	Leu 154: O	Hydrogen Bond	2.21	−2.75
		Trp 156–Argpyrimidine	Trp 156: H	Argpyrimidine: O	Hydrogen Bond	2.38	
		Glu 174–Argpyrimidine	Argpyrimidine: H	Glu 174: O	Hydrogen Bond	1.82	
		Glu 174–Argpyrimidine	Argpyrimidine: N	Glu 174: O	Electrostatic Interaction	2.82	

**Table 3:** The list of *Rattus norvegicus* primers used for quantification of mRNA using qRT-PCR.

Sr. No.	Name of the Gene	Sense Primer Sequence	Antisense Primer Sequence
1.	18s	5′ACGGAAGGGCACCACCAGGA 3′	5′CACCACCACCCACGGAATCG 3′
2.	RAGE	5′ GGTACTGGTTCTTGCTCT 3′	5′ATTCTAGCTTCTGGGTTG 3′
3.	TNF-α	5′ CAAGGAGGAGAAGTTCCCAA 3′	5′CTCTGCTTGGTGGTTTGCTA 3′
4.	ICAM-1	5′CCCCACCTACATACATTCCTAC 3′	5′ACATTTTCTCCCAGGCATTC 3′
5.	NADPH oxidase	5′ GGCATCCCTTTACTCTGACCT 3′	5′ TGCTGCTCGAATATGAATGG 3′
6.	IL-6	5′ GCCCTTCAGGAACAGCTATGA 3′	5′ TGTCAACAACATCAGTCCCAAGA 3′
7.	IL-1β	5′ CCCTGCAGCTGGAGAGTGTGG 3′	5′ TGTGCTCTGCTTGAGAGGTGCT 3′
8.	TGF-β	5′ TGCTTCAGCTCCACAGAGAA 3′	5′ TGTGTTGGTTGTAGAGGGCA 3′
9.	iNOS	5′ TCACTGGGACAGCACAGAAT 3′	5′ TGTGTCTGCAGATGTGCTGA 3′
10.	Fibronectin	5′ CATGGCTTTAGGCGAACCA 3′	5′ CATCTACATTCGGCAGGTATGG 3′
11.	*α*-SMA	5′ GACCCTGAAGTATCCGATAGAACA 3′	5′CACGCGAAGCTCGTTATAGAAG 3′
12.	E-cadherin	5′ TGATGATGCCCCCAACACTC 3′	5′ CCAAGCCCTTGGCTGTTTTC 3′
13.	p38 MAPK	5′ CGAAATGACCGGCTACGTGG 3′	5′ CACTTCATCGTAGGTCAGGC 3′
14.	Grp78	5′ GAAACTGCCGAGGCGTAT 3′	5′ ATGTTCTTCTCTCCCTCTCTCTTT 3′
15.	CHOP	5′ GAAAGCAGAAACCGGTCCAAT 3′	5′ GGATGAGATATAGGTGCCCCC 3′
16.	MCP-1	5′ CCTCCACCACTATGCAGGTCTC 3′	5′ GCACGTGGATGCTACAGGC 3′
17.	Nrf-2	5′ CAGAGTTTCTTCGCCAGAGG 3′	5′ TGAGTGTGAGGACCCATCG 3′
18.	HO-1	5′ CAAATCCCACCTTGAACACA 3′	5′ CGACTGACTAATGGCAGCAG 3′

## Data Availability

All data generated or analyzed during the current study are included in the manuscript.

## Supplementary Materials

The following are available online, Figure S1: The stages involved in the formation of advanced glycation end products.

## References

[1] (2003). Writing Team for the Diabetes Control and Complications Trial/Epidemiology of Diabetes Interventions and Complications Research Group Sustained Effect of Intensive Treatment of Type 1 Diabetes Mellitus on Development and Progression of Diabetic Nephropathy. JAMA.

[2] Fioretto P., Steffes M.W., Sutherland D.E., Goetz F.C., Mauer M. (1998). Reversal of Lesions of Diabetic Nephropathy after Pancreas Transplantation. N. Engl. J. Med..

[3] Barbosa J., Steffes M.W., E Sutherland D., E Connett J., Rao K.V., Mauer S.M. (1994). Effect of glycemic control on early diabetic renal lesions. A 5-year randomized controlled clinical trial of insulin-dependent diabetic kidney transplant recipients. JAMA.

[4] Yao D., Brownlee M. (2009). Hyperglycemia-Induced Reactive Oxygen Species Increase Expression of the Receptor for Advanced Glycation End Products (RAGE) and RAGE Ligands. Diabetes.

[5] Neglia C., Cohen H.J., Garber A.R., Ellis P.D., Thorpe S.R., Baynes J.W. (1983). 13C NMR investigation of nonenzymatic glucosylation of protein. Model studies using RNase A. J. Biol. Chem..

[6] Lapolla A., Traldi P., Fedele D. (2005). Importance of measuring products of non-enzymatic glycation of proteins. Clin. Biochem..

[7] Kilhovd B., Giardino I., Torjesen P., Birkeland K., Berg T., Thornalley P., Brownlee M., Hanssen K. (2003). Increased serum levels of the specific AGE-compound methylglyoxal-derived hydroimidazolone in patients with type 2 diabetes. Metabolism.

[8] Cooper M.E. (2001). Interaction of metabolic and haemodynamic factors in mediating experimental diabetic nephropathy. Diabetologia.

[9] Pasupulati A.K., Chitra P.S., Reddy G.B. (2016). Advanced glycation end products mediated cellular and molecular events in the pathology of diabetic nephropathy. Biomol. Concepts.

[10] Ceriello A., Bortolotti N., Falleti E., Taboga C., Tonutti L., Crescentini A., Motz E., Lizzio S., Russo A., Bartoli E. (1997). Total Radical-Trapping Antioxidant Parameter in NIDDM Patients. Diabetes Care.

[11] Kang J.H. (2003). Modification and inactivation of human Cu,Zn-superoxide dismutase by methylglyoxal. Mol. Cells.

[12] Najjar F.M., Taghavi F., Ghadari R., Sheibani N., Moosavi-Movahedi A.A. (2017). Destructive effect of non-enzymatic glycation on catalase and remediation via curcumin. Arch. Biochem. Biophys..

[13] Choudhary M.I., Maher S., Begum A., Abbaskhan A., Ali S., Khan A., Rehman S.-U.-, Rahman A.-U. (2010). Characterization and Antiglycation Activity of Phenolic Constituents from Viscum album (European Mistletoe). Chem. Pharm. Bull..

[14] Meng G., Zhu H., Yang S., Wu F., Zheng H., Chen E., Xu J. (2011). Attenuating effects of Ganoderma lucidum polysaccharides on myocardial collagen cross-linking relates to advanced glycation end product and antioxidant enzymes in high-fat-diet and streptozotocin-induced diabetic rats. Carbohydr. Polym..

[15] Sun Z., Peng X., Liu J., Fan K.W., Wang M., Chen F. (2010). Inhibitory effects of microalgal extracts on the formation of advanced glycation endproducts (AGEs). Food Chem..

[16] Yazdanparast R., Ardestani A., Jamshidi S. (2007). Experimental diabetes treated with Achillea santolina: Effect on pancreatic oxidative parameters. J. Ethnopharmacol..

[17] Murali B., Upadhyaya U., Goyal R. (2002). Effect of chronic treatment with Enicostemma littorale in non-insulin-dependent diabetic (NIDDM) rats. J. Ethnopharmacol..

[18] Yamahara J., Kobayashi M., Matsuda H., Aoki S. (1991). Anticholinergic action of Swertia japonica and an active constituent. J. Ethnopharmacol..

[19] Vaidya H., Rajani M., Sudarsanam V., Padh H., Goyal R. (2009). Antihyperlipidaemic activity of swertiamarin, a secoiridoid glycoside in poloxamer-407-induced hyperlipidaemic rats. J. Nat. Med..

[20] Vaidya H., Prajapati A., Rajani M., Sudarsanam V., Padh H., Goyal R.K. (2012). Beneficial Effects of Swertiamarin on Dyslipidaemia in Streptozotocin-induced Type 2 Diabetic Rats. Phytotherapy Res..

[21] Jaishree V., Badami S. (2010). Antioxidant and hepatoprotective effect of swertiamarin from Enicostemma axillare against d-galactosamine induced acute liver damage in rats. J. Ethnopharmacol..

[22] Jaishree V., Narsimha S. (2020). Swertiamarin and quercetin combination ameliorates hyperglycemia, hyperlipidemia and oxidative stress in streptozotocin-induced type 2 diabetes mellitus in wistar rats. Biomed. Pharmacother..

[23] Nakagawa T., Yokozawa T., Terasawa K., Shu S., Juneja L.R. (2002). Protective Activity of Green Tea against Free Radical- and Glucose-Mediated Protein Damage. J. Agric. Food Chem..

[24] Vishwakarma S., Rajani M., Bagul M., Goyal R. (2004). A Rapid Method for the Isolation of Swertiamarin from Enicostemma littorale. Pharm. Biol..

[25] Magora H.B., Rahman M., Gray A.I., Cole M.D. (2003). Swertiamarin from Enicostemma axillare subsp. axillare (Gentianaceae). Biochem. Syst. Ecol..

[26] Rana V.S. (2014). Separation and Identification of Swertiamarin from Enicostema axillare Lam. Raynal by Centrifugal Partition Chromatography and Nuclear Magnetic Resonance-Mass Spectrometry. J. Pharm Sci. Emerg. Drugs.

[27] Rana V.S., Dhanani T., Kumar S. (2012). Improved and Rapid HPLC-PDA Method for Identification and Quantification of Swertiamarin in the Aerial Parts of Enicostemma Axillare. Malaysian J. Pharma Sci..

[28] Kumar S., Jairaj V. (2018). An Effective Method for Isolation of Pure Swertiamarin from Enicostemma littorale Blume. Indo Glob. J. Pharm. Sci..

[29] Wu T., Li J., Li Y., Song H. (2017). Antioxidant and Hepatoprotective Effect of Swertiamarin on Carbon Tetrachloride-Induced Hepatotoxicity via the Nrf2/HO-1 Pathway. Cell. Physiol. Biochem..

[30] Nita M., Grzybowski A. (2016). The Role of the Reactive Oxygen Species and Oxidative Stress in the Pathomechanism of the Age-Related Ocular Diseases and Other Pathologies of the Anterior and Posterior Eye Segments in Adults. Oxidative Med. Cell. Longev..

[31] Fukami K., Yamagishi S.-I., Ueda S., Okuda S. (2008). Role of AGEs in Diabetic Nephropathy. Curr. Pharm. Des..

[32] Ahmed N. (2005). Advanced glycation endproducts—role in pathology of diabetic complications. Diabetes Res. Clin. Pract..

[33] Singh V.P., Bali A., Singh N., Jaggi A.S. (2014). Advanced Glycation End Products and Diabetic Complications. Korean J. Physiol. Pharmacol..

[34] Lunceford N., Gugliucci A. (2005). Ilex paraguariensis extracts inhibit AGE formation more efficiently than green tea. Fitoter..

[35] Lapolla A., Flamini R., Vedova A.D., Senesi A., Reitano R., Fedele D., Basso E., Seraglia R., Traldi P. (2003). Glyoxal and Methylglyoxal Levels in Diabetic Patients: Quantitative Determination by a New GC/MS Method. Clin. Chem. Lab. Med..

[36] Stinghen A.E., Massy Z.A., Vlassara H., Striker G.E., Boullier A. (2015). Uremic Toxicity of Advanced Glycation End Products in CKD. J. Am. Soc. Nephrol..

[37] Henning C., Liehr K., Girndt M., Ulrich C., Glomb M.A. (2014). Extending the Spectrum of α-Dicarbonyl Compounds In Vivo. J. Biol. Chem..

[38] Wang W., Yagiz Y., Buran T.J., Nunes C.D.N., Gu L. (2011). Phytochemicals from berries and grapes inhibited the formation of advanced glycation end-products by scavenging reactive carbonyls. Food Res. Int..

[39] Justino A.B., Franco R.R., Silva H.C.G., Saraiva A.L., Sousa R.M.F., Espindola F.S. (2019). B procyanidins of Annona crassiflora fruit peel inhibited glycation, lipid peroxidation and protein-bound carbonyls, with protective effects on glycated catalase. Sci. Rep..

[40] Ardestani A., Yazdanparast R. (2007). Cyperus rotundus suppresses AGE formation and protein oxidation in a model of fructose-mediated protein glycoxidation. Int. J. Biol. Macromol..

[41] Ahmad S., Shahab U., Baig M.H., Khan M.S., Khan M.S., Srivastava A.K., Saeed M. (2013). Moinuddin Inhibitory Effect of Metformin and Pyridoxamine in the Formation of Early, Intermediate and Advanced Glycation End-Products. PLoS ONE.

[42] Ishibashi Y., Matsui T., Takeuchi M., Yamagishi S. (2012). Metformin Inhibits Advanced Glycation End Products (AGEs)-induced Renal Tubular Cell Injury by Suppressing Reactive Oxygen Species Generation via Reducing Receptor for AGEs (RAGE) Expression. Horm. Metab. Res..

[43] Meeprom A., Sompong W., Chan C.B., Adisakwattana S. (2013). Isoferulic Acid, a New Anti-Glycation Agent, Inhibits Fructose- and Glucose-Mediated Protein Glycation In Vitro. Molecules.

[44] Jairajpuri D.S., Fatima S., Saleemuddin M. (2007). Immunoglobulin glycation with fructose: A comparative study. Clin. Chim. Acta.

[45] Allarakha S., Ahmad P., Ishtikhar M., Zaheer M.S., Siddiqi S.S., Moinuddin, Ali A. (2015). Fructosylation generates neo-epitopes on human serum albumin. IUBMB Life.

[46] Liu J., Xing X., Jing H. (2018). Differentiation of glycated residue numbers on heat-induced structural changes of bovine serum albumin. J. Sci. Food Agric..

[47] Yan H., Harding J.J. (1997). Glycation-induced inactivation and loss of antigenicity of catalase and superoxide dismutase. Biochem. J..

[48] Ha H., Hwang I.-A., Park J.H., Lee H.B. (2008). Role of reactive oxygen species in the pathogenesis of diabetic nephropathy. Diabetes Res. Clin. Pract..

[49] Liu B.-F., Miyata S., Hirota Y., Higo S., Miyazaki H., Fukunaga M., Hamada Y., Ueyama S., Muramoto O., Uriuhara A. (2003). Methylglyoxal induces apoptosis through activation of p38 mitogen-activated protein kinase in rat mesangial cells. Kidney Int..

[50] Oya T., Hattori N., Mizuno Y., Miyata S., Maeda S., Osawa T., Uchida K. (1999). Methylglyoxal Modification of Protein. J. Biol. Chem..

[51] Wilker S.C., Chellan P., Arnold B.M., Nagaraj R.H. (2001). Chromatographic Quantification of Argpyrimidine, a Methylglyoxal-Derived Product in Tissue Proteins: Comparison with Pentosidine. Anal. Biochem..

[52] Padival A.K., Crabb J.W., Nagaraj R.H. (2003). Methylglyoxal modifies heat shock protein 27 in glomerular mesangial cells. FEBS Lett..

[53] Niki E. (2008). Lipid peroxidation products as oxidative stress biomarkers. BioFactors.

[54] Ayala A., Muñoz M.F., Argüelles S. (2014). Lipid Peroxidation: Production, Metabolism, and Signaling Mechanisms of Malondialdehyde and 4-Hydroxy-2-Nonenal. Oxid. Med. Cell. Longev..

[55] Sompong W., Cheng H., Adisakwattana S. (2016). Ferulic acid prevents methylglyoxal-induced protein glycation, DNA damage, and apoptosis in pancreatic β-cells. J. Physiol. Biochem..

[56] Reiniger N., Lau K., McCalla D., Eby B., Cheng B., Lu Y., Qu W., Quadri N., Ananthakrishnan R., Furmansky M. (2010). Deletion of the Receptor for Advanced Glycation End Products Reduces Glomerulosclerosis and Preserves Renal Function in the Diabetic OVE26 Mouse. Diabetes.

[57] Ye S.D., Zheng M., Zhao L.L., Qian Y., Yao X.M., Ren A., Li S.M., Jing C.Y. (2009). Intensive insulin therapy decreases urinary MCP-1 and ICAM-1 excretions in incipient diabetic nephropathy. Eur. J. Clin. Investig..

[58] Yamagishi S.-I., Matsui T. (2010). Advanced Glycation end Products, Oxidative Stress and Diabetic Nephropathy. Oxidative Med. Cell. Longev..

[59] Menon M.C., Ross M.J. (2016). Epithelial-to-mesenchymal transition of tubular epithelial cells in renal fibrosis: A new twist on an old tale. Kidney Int..

[60] Zhang X., Liang D., Guo L., Liang W., Jiang Y., Li H., Zhao Y., Lu S., Chi Z.-H. (2012). Curcumin protects renal tubular epithelial cells from high glucose-induced epithelial-to-mesenchymal transition through Nrf2-mediated upregulation of heme oxygenase-1. Mol. Med. Rep..

[61] Burns W.C., Twigg S.M., Forbes J.M., Pete J., Tikellis C., Thallas-Bonke V., Thomas M.C., Cooper M.E., Kantharidis P. (2006). Connective Tissue Growth Factor Plays an Important Role in Advanced Glycation End Product–Induced Tubular Epithelial-to-Mesenchymal Transition: Implications for Diabetic Renal Disease. J. Am. Soc. Nephrol..

[62] Lv Z.-M., Wang Q., Wan Q., Lin J.-G., Hu M.-S., Liu Y.-X., Wang R. (2011). The Role of the p38 MAPK Signaling Pathway in High Glucose-Induced Epithelial-Mesenchymal Transition of Cultured Human Renal Tubular Epithelial Cells. PLoS ONE.

[63] Lee Y.J., Han H.J. (2010). Troglitazone ameliorates high glucose-induced EMT and dysfunction of SGLTs through PI3K/Akt, GSK-3β, Snail1, and β-catenin in renal proximal tubule cells. Am. J. Physiol. Physiol..

[64] Hung T.-J., Chen W.-M., Liu S.-F., Liao T.-N., Lee T.-C., Chuang L.-Y., Guh J.-Y., Hung C.-Y., Hung Y.-J., Chen P.-Y. (2012). 20-Hydroxyecdysone attenuates TGF-β1-induced renal cellular fibrosis in proximal tubule cells. J. Diabetes Complicat..

[65] Parwani K., Mandal P. (2020). Role of advanced glycation end products and insulin resistance in diabetic nephropathy. Arch. Physiol. Biochem..

[66] Lu Q., Wang W., Zhang M., Ma Z., Qiu X., Shen M., Yin X. (2018). ROS induces epithelial-mesenchymal transition via the TGF-β1/PI3K/Akt/mTOR pathway in diabetic nephropathy. Exp. Ther. Med..

[67] Jiang T., Huang Z., Lin Y., Zhang Z., Fang D., Zhang D.D. (2010). The Protective Role of Nrf2 in Streptozotocin-Induced Diabetic Nephropathy. Diabetes.

[68] Kie J.H., Kapturczak M.H., Traylor A., Agarwal A., Hill-Kapturczak N. (2008). Heme Oxygenase-1 Deficiency Promotes Epithelial-Mesenchymal Transition and Renal Fibrosis. J. Am. Soc. Nephrol..

[69] Fatchiyah F., Hardiyanti F., Widodo N. (2015). Selective Inhibition on RAGE-binding AGEs Required by Bioactive Peptide Alpha-S2 Case in Protein from Goat Ethawah Breed Milk: Study of Biological Modeling. Acta Inform. Medica.

[70] Kshirsagar P.R., Pai S.R., Nimbalkar M.S., Gaikwad N.B. (2016). RP-HPLC analysis of seco-iridoid glycoside swertiamarin from differentSwertiaspecies. Nat. Prod. Res..

[71] McPherson J.D., Shilton B.H., Walton D.J. (1988). Role of fructose in glycation and cross-linking of proteins. Biochemistry.

[72] Riss T.L., Moravec R.A., Niles A.L., Duellman S., Benink H.A., Worzella T.J., Minor L. (2004). Assay Guidance Manual.

[73] Karbowski M., Kurono C., Wozniak M., Ostrowski M., Teranishi M., Nishizawa Y., Usukura J., Soji T., Wakabayashi T. (1999). Free radical–induced megamitochondria formation and apoptosis. Free. Radic. Biol. Med..

[74] Tukozkan N., Erdamar H., Seven I. (2006). Measurement of total malondialdehyde in plasma and tissues by high-performance liquid chromatography and thiobarbituric acid assay. Firat Tip Dergisi..

[75] Kema V.H., Khan I., Jamal R., Vishwakarma S.K., Reddy C.L., Parwani K., Patel F., Patel D., Khan A.A., Mandal P. (2017). Protective Effects of Diallyl Sulfide Against Ethanol-Induced Injury in Rat Adipose Tissue and Primary Human Adipocytes. Alcohol. Clin. Exp. Res..

[76] Tsoporis J., Izhar S., Leong-Poi H., Desjardins J.-F., Huttunen H., Parker T. (2010). S100B Interaction with the Receptor for Advanced Glycation End Products (RAGE). Circ. Res..

[77] Apweiler R. (2004). UniProt: The Universal Protein knowledgebase. Nucleic Acids Res..

[78] Xu D., Young J.H., Krahn J.M., Song D., Corbett K.D., Chazin W.J., Pedersen L.C., Esko J.D. (2013). Stable RAGE-Heparan Sulfate Complexes Are Essential for Signal Transduction. ACS Chem. Biol..

[79] (2021). Schrödinger Release, Version 12.7.156.

[80] Sastry G.M., Adzhigirey M., Day T., Annabhimoju R., Sherman W. (2013). Protein and ligand preparation: Parameters, protocols, and influence on virtual screening enrichments. J. Comput. Mol. Des..

[81] Greenwood J.R., Calkins D., Sullivan A.P., Shelley J.C. (2010). Towards the comprehensive, rapid, and accurate prediction of the favorable tautomeric states of drug-like molecules in aqueous solution. J. Comput. Mol. Des..

[82] Jacobson M.P., Pincus D.L., Rapp C.S., Day T.J.F., Honig B., Shaw D.E., Friesner R.A. (2004). A hierarchical approach to all-atom protein loop prediction. Proteins: Struct. Funct. Bioinform..

