# Supplementary material for: Protective Effects of Swertiamarin against Methylglyoxal-Induced Epithelial-Mesenchymal Transition by Improving Oxidative Stress in Rat Kidney Epithelial (NRK-52E) Cells
Source: Molecules. 2021 May 7;26(9):2748. doi: 10.3390/molecules26092748 (PMC8125635; doi:10.3390/molecules26092748)
Supplement: Supplementary file 1 [file molecules-26-02748-s001.zip › molecules-1160615-Supplementary.pdf]

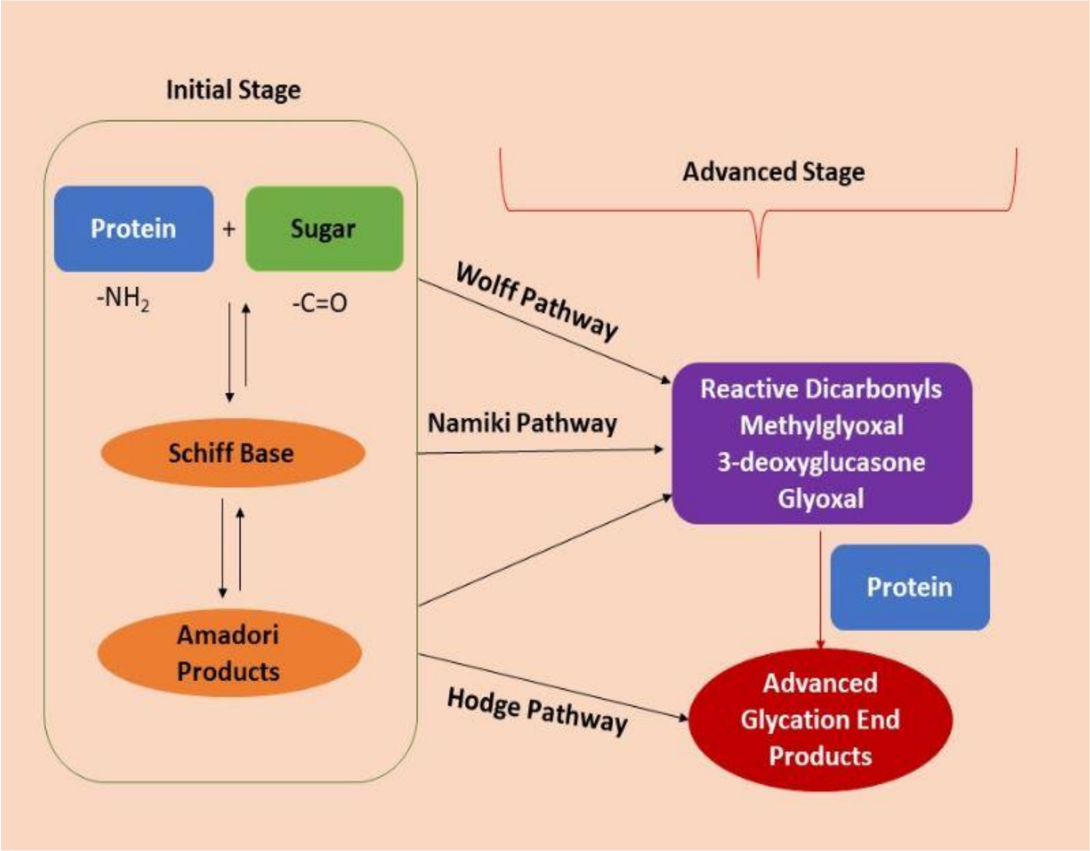

Supplementary figure 1: The stages involved in the formation of advanced glycation end products.
